# Assessment of PSA-Age volume score in predicting positive prostate biopsy findings in Turkey

**DOI:** 10.1590/S1677-5538.IBJU.2014.0462

**Published:** 2015

**Authors:** Oktay Uçer, Uğur Yücetaş, İlker Çelen, Gökhan Toktaş, Talha Müezzinoğlu

**Affiliations:** 1 Department of Urology, Faculty of Medicine, Celal Bayar University, Manisa, Turkey; 2 Department of Urology, Istanbul Teaching and Research Hospital, Istanbul, Turkey; 3 Urology Clinic, Acipayam State Hospital, Denizli, Turkey

**Keywords:** Prostate-Specific Antigen, Prostatic Neoplasms, Prostate, Biopsy, Digital Rectal Examination

## Abstract

**Objectives:**

To evaluate PSA-age volume (AV) scores in predicting positive prostate biopsy findings in Turkey.

**Materials and Methods:**

PSA-AV was calculated by multiplying the patient's age by the prostate volume and dividing it by the PSA level. Sensitivities and specificities of the PSA-AV were assessed by retrospective analysis of findings from 4,717 prostate biopsies.

**Results:**

The population's average age was 63.71±7.63 years, the mean PSA level was 9.73±17.01ng/mL, the mean prostate volume was 44.46±23.88 cm^3^. Of the 4,717 prostate biopsies, 1,171 biopsy specimens (24.8%) were positive for prostate cancer. A PSA-AV score of 700 had a sensitivity and specificity of 95% and 15%, respectively. These values were similar to the sensitivity and specificity for a PSA cut-off of 4ng/mL (94% and 13%, respectively). Although the sensitivity of a PSA-AV cut-off of 700 in patients over 60 years was similar to the PSA cut-off of 4ng/mL and the age-adjusted PSA, in patients <60 years, its sensitivity was higher. While the sensitivities of a PSA-AV cut-off of 700 in patients with low prostate volume was higher than a PSA cut-off of 4ng/ mL, the sensitivities of both methods with moderate prostate volumes were similar.

**Conclusions:**

Considering all the biopsies, the sensitivity and specificity of a PSA-AV of 700 for predicting positive biopsy findings were similar to a PSA of 4ng/mL. We suggest the PSA-AV cut-off of 700 should only be used in patients younger than 60 with low prostate volumes (<20cm^3^).

## INTRODUCTION

Regular serum prostate-specific antigen (PSA) evaluations and digital rectal examinations (DRE) are recommended for detecting PCa (1). However, the serum PSA level is the most widely used marker to detect this cancer in the general population. Because PSA is organ specific, but not disease specific, its use for prostate cancer screening lacks adequate sensitivity (2). Thus, due to the false-positive results obtained by the PSA test during screening, many patients are subjected to an unnecessary transrectal ultrasound-guided prostatic biopsy (TRUSPB), which is an invasive procedure that can lead to significant morbidity, and even mortality (3, 4).

Recently, various strategies were introduced to improve the sensitivity and specificity of the PSA (5). In these studies, prostate volume, PSA level and age were clinically significant predictors of positive biopsy findings (5, 6). Patel et al. (7) developed a novel formula that incorporates age, prostate volume, race and PSA level into a single score for prostate cancer detection. The PSA-age volume (PSA-AV) score is calculated by multiplying the age and prostate volume and then dividing the total by the pre-biopsy PSA. Patel et al. noticed the formula is useful for predicting positive biopsy findings. According to their data, the PSA-AV score was more sensitive in younger patients and in patients with a small prostate volume. They also reported that the PSA-AV score was more specific in older patients and in patients with a large prostate volume. The purpose of the present retrospective study was to evaluate the novel score in predicting positive prostate biopsy findings in Turkey.

## MATERIALS AND METHODS

This retrospective study was based on the data of 5,299 TRUSPB procedures performed between 2005 and 2013 at the Department of Urology of the University Hospital in Manisa and the Clinic of Urology of the Teaching and Research Hospital in Istanbul, Turkey. The indications for performing a TRUSPB were elevated or increasing PSA levels, abnormal DRE findings or a previous abnormal TRUSPB. The database consisted of four variables including age, pre-biopsy PSA level, prostate volume and digital rectal examination information.

TRUSPBs were performed using the LOGIQ machine at both the University Hospital and the Teaching and Research Hospital by the urologists. The prostate volume was calculated using ultrasonography during the TRUSPB. The number of biopsy cores (6-12 cores) was determined by the urologists according to their preference. In patients with an abnormal DRE or ultrasound findings, additional biopsy cores were taken.

A total of 5,063 biopsy records were reviewed. We eliminated those biopsy records that did not have complete data, number of biopsy cores with less than 10, patients who were <40 years or >79 years old, and patients who had undergone repeat biopsies. A total of 4,717 biopsy specimens were analysed. The PSA-age volume (PSA-AV) score was calculated by multiplying the age and prostate volume and then dividing the total by the pre-biopsy PSA level (7).

Patel et al. (7) stratified the PSA-AV score in intervals of 400, and also analysed the score to determine an effective cut-off score for predicting prostate cancer. The sensitivities and specificities of the PSA AV of 500 and 700 were also analysed since the PSA-AV of 500 or 700 were recommended as a PSA-AV cut-off in their study. Therefore we used PSA-AV of 500 and 700 as cut-off of PSA-AV. The sensitivities and specificities of PSA-AV of 500 and 700 were calculated in the patient groups divided according to age and prostate volume. The sensitivities and specificities of the age-adjusted PSA levels and PSA cut-off of 4ng/mL were calculated. For the age-adjusted PSA data, patients were categorized into four categories, each with its own abnormal PSA value. The abnormal values were >2.5ng/mL (age 40-49 years), >3.5ng/mL (age 50-59 years), >4.5ng/mL (age 60-69 years), and >6.5ng/mL (age 70-79 years). Statistical analyses were carried out using SPSS 13.0 (SPSS Inc.). The study protocol was approved by the ethics committee of our institution.

## RESULTS

The mean age of the patients in our study was 63.71±7.63 (n=4,717). The mean PSA level and mean prostate volume were 9.73±17.01ng/mL and 44.46±23.88cm^3^, respectively (10% trimmed mean). Of the 4,717 prostate biopsies, 1,171 biopsy specimens (24.8%) were positive for prostate cancer.

The sensitivities and specificities of the PSA-AV scores in intervals of 400, PSA-AV of 500 and 700, and PSA cut-off of 4ng/mL are shown in Figure-1. The positive predictive value of the PSA-AV cut-off of 500 and 700 was 30% and 27%, respectively. The positive predictive value of the PSA cut-off of 4ng/mL and the age-adjusted PSA method was 26% and 25%, respectively. Although using a PSA-AV cut-off of 700 decreased the number of biopsies by 114, it led to 10 more detected cancer cases compared to using the PSA cut-off of 4ng/mL. In the same population, using a PSA cut-off of 4ng/mL increased the biopsies taken by 875 compared with a PSA-AV cut-off of 500 and led to 95 more detected cancer cases. The sensitivity, specificity, positive predictive and negative predictive value changes within the age and prostate volume groups are listed in Tables 1 and 2.

**Table 1:** Sensitivity and specificity of various cut-off methods in different age groups.

Variable		Total biopsies (n)	Cancers detected (n)	Sensitivity (%)	Specificity (%)	PPV (%)	NPV (%)
**Age 40-49 years**							
	Psa cut off 2.5ng/mL	120	12	85.7	6.1	10.0	77.8
	Psa cut off 4.0ng/mL	91	9	64.3	28.7	9.9	86.8
	Psaav cut off 700	123	14	100	5.2	11.4	100
	Psaav cut off 500	108	13	92.9	17.4	12.0	95.2
**50-59 years**							
	Psa cut off 3.5ng/mL	1191	199	91.7	12.0	16.7	88.2
	Psa cut off 4.0ng/mL	1081	192	88.5	21.1	17.8	90.5
	Psaav cut off 700	1224	213	98.2	10.3	17.4	96.7
	Psaav cut off 500	1005	194	89.4	28	19.3	93.2
**60-69 years**							
	Psa cut off 4.5ng/mL	1767	445	89.6	15.9	25.2	82.8
	Psa cut off 4.0ng/mL	1908	472	95.0	8.7	24.7	84.5
	Psaav cut off 700	1764	474	95.5	17.9	26.9	92.5
	Psaav cut off 500	1408	424	85.3	37.4	30.1	89.0
**70-79 years**							
	Psa cut off 6.5ng/mL	912	375	84.7	26.6	44.1	74.1
	Psa cut off 4.0ng/mL	1128	430	97.1	4.6	38.1	72.3
	Psaav cut off 700	983	412	93.0	22.0	41.9	83.9
	Psaav cut off 500	812	377	85.1	40.6	46.4	81.8

**PPV** = Positive predictive value; **NPV** = Negative predictive value; **PSA** = prostate-specific antigen; **PSA-AV** = PSA-age volume.

**Table 2:** Sensitivity and specificity of PSA-AV cut-off of 700 and 500, and PSA cut-off of 4ng/mL in different prostate volume groups.

Variable		Total biopsies (n)	Cancers detected (n)	Sensitivity (%)	Specificity (%)	PPV (%)	NPV (%)
**Prostate volume <20cm^3^**							
	Psa cut off 4.0ng/mL	298	139	90.8	25.4	46.6	79.4
	Psaav cut off 700	345	151	98.7	8.9	43.8	90.5
	Psaav cut off 500	341	151	98.7	6.3	44.3	92.0
**Prostate volume 20-60cm^3^**							
	Psa cut off 4.0ng/mL	2914	821	94.0	14.2	28.2	86.9
	Psaav cut off 700	3072	849	97.3	8.8	27.6	90.0
	Psaav cut off 500	2555	778	89.1	27.1	30.5	87.4
**Prostate volume 60-100cm^3^**							
	Psa cut off 4.0ng/mL	786	116	100.0	5.4	14.8	100.0
	Psaav cut off 700	553	91	78.4	34.7	16.5	90.8
	Psaav cut off 500	358	63	54.3	58.3	17.6	88.6

**PPV** = Positive predictive value; **NPV** = Negative predictive value; **PSA** = prostate-specific antigen; **PSA-AV** = PSA-age volume.

## DISCUSSION

Significant research efforts are ongoing to identify the optimal PSA threshold to recommend a prostate biopsy in an asymptomatic patient (5, 8). Catalona et al. reported this PSA level or higher was appropriate as the PSA cut-off value for the screening of PCa. Since then, this value has been the most commonly used clinically. While its sensitivity is 67.5% to 80%, the specificity is only 20% to 30% (9). Although PSA is a highly organspecific marker, it is not a cancer-specific marker. Therefore, it may also show increases with age or other benign conditions, including benign prostate hyperplasia or prostate inflammation (10). Prostate-specific antigen density (PSAD) was investigated to decrease the impact of prostate volume on the PSA level before deciding on a TRUSPB. Because studies using PSAD for prostate cancer screening have led to conflicting results, it is not widely used by clinicians (11). PSA-AV was developed by Patel et al. to correct the impact of prostate volume on PSA levels (7). They noticed that a PSA-AV score of 700 was a useful formula for predicting positive biopsy findings in patients with small prostates. According to their data, in patients with low to moderate prostate volumes (<20cm^3^ and 20-60cm^3^), a PSA-AV cut-off of 700 had sensitivities of 97% and 91%, respectively compared with sensitivities of 74% and 86% for a PSA cut-off of 4ng/mL. This makes it useful for ruling out prostate cancer. In patients with low to moderate prostate volumes (<20cm^3^ and 20-60cm^3^) in our study, a PSA-AV cut-off of 700 had sensitivities of 98% and 97%, respectively. The sensitivities of the PSA cut-off of 4ng/mL were 91% and 94%, respectively, in the same patient group. In both studies, the sensitivities of the PSA-AV cut-off of 700 were higher than the sensitivities of the PSA cut-off of 4ng/mL. However, the sensitivities of the PSA cut-off of 4ng/mL in our study were higher compared with their study. Although their study had 687 patients (prostate volume <60cm^3^) receiving a TRUSPB, our study had 3,417 patients. Therefore, we think that our data is statistically more reliable. Our findings suggest that, although the sensitivities of PSA-AV cut-off of 700 in patients with low prostate volumes (<20cm^3^) are higher than with a PSA cut-off of 4ng/mL, the sensitivities of the two methods in patients with moderate prostate volumes (20-60cm^3^) are similar.

Considering all of the biopsies, we found that the sensitivities of a PSA-AV cut-off of 700 and a PSA cut-off of 4ng/mL were 95% and 94%, respectively. Positive predictive values of a PSA-AV cut-off of 700 and a PSA cut-off of 4ng/mL were 27% and 26%, respectively. According to our data, the effectiveness of the PSA-AV cut-off of 700 compared with a PSA cut-off of 4ng/mL are similar for predicting positive biopsy findings, considering all age groups and prostate volumes. Compared with using the PSA cut-off of 4ng/mL, using the PSAAV of 700 decreased the number of biopsies by 114; however, it detected 45 more cancer cases. Patel et al. (7) noticed that using a PSA-AV cut-off of 700, rather than a PSA cut-off of 4ng/mL, led to 16 fewer biopsies with seven additional cancers detected.

Our data showed that in the 60-69-year-old population, the sensitivities of an age-adjusted PSA, a PSA cut-off of 4ng/mL and a PSA-AV cut-off of 700 were 90%, 95% and 95%, respectively. In this age group, the diagnostic values of these three methods for predicting positive prostate biopsy findings are similar to each other. In patients younger than 60, the sensitivity of the PSA-AV cut-off of 700 was higher than the age-adjusted PSA and the PSA cut-off of 4ng/mL (Table-1). In the 50-59-year-old population, the sensitivities of the PSA cut-off of 4ng/mL, the PSA cut-off of 3.5ng/ mL and a PSA-AV cut-off of 700 were 88%, 91% and 98%, respectively. In this group, compared with using the PSA cut-off of 4ng/mL, using PSA-AV cut-off of 700 led to 143 more biopsies and 21 more cancer cases detected. Although the effectiveness of a PSA-AV cut-off of 700 in patients aged over 60 is similar to the other methods, in patients under 60 years old its effectiveness seems higher. Similarly, Patel et al. (7) suggest that a PSA-AV score of 700 is useful in ruling out cancer in younger patients.

US Preventive Services Task Force noticed that the amount of overdiagnosis of prostate cancer is an important concern because a man with cancer that would remain asymptomatic for the remainder of his life cannot benefit from screening or treatment (12). One of the limitations of our study was that we did not divide the patients into groups according to their Gleason score. Therefore we could not assess overdiagnosis of PCa and insignificant cancer in our study population. We suggest that further studies should evaluate the effect of PSA-AV formula on insignificant prostate cancer and overdiagnosis.

## CONCLUSIONS

Our data supports the findings from the previous study that developed PSA-AV formula. However, in patients with a moderate prostate volume (20-60cm^3^), we did not determine any superiority of the PSA-AV formula. Therefore, we suggest that the PSA-AV cut-off of 700 be used for predicting positive prostate biopsy findings in patients under the age of 60 and with low prostate volumes (<20cm^3^). Further studies should evaluate the effectiveness of PSA and PSA-AV for predicting positive prostate biopsy findings in the patients without abnormal DRE.
